# Astrocytes Do Not Forfeit Their Neuroprotective Roles After Surviving Intense Oxidative Stress

**DOI:** 10.3389/fnmol.2019.00087

**Published:** 2019-04-05

**Authors:** Tarun N. Bhatia, Deepti B. Pant, Elizabeth A. Eckhoff, Rachel N. Gongaware, Timothy Do, Daniel F. Hutchison, Amanda M. Gleixner, Rehana K. Leak

**Affiliations:** Graduate School of Pharmaceutical Sciences, Duquesne University, Pittsburgh, PA, United States

**Keywords:** astrocyte, stress response, oxidative, paraquat, preconditioning, glia, Parkinson’s disease

## Abstract

In order to fulfill their evolutionary role as support cells, astrocytes have to tolerate intense oxidative stress under conditions of brain injury and disease. It is well known that astrocytes exposed to mild oxidative stress are preconditioned against subsequent stress exposure in dual hit models. However, it is unclear whether *severe* oxidative stress leads to stress tolerance, stress exacerbation, or no change in stress resistance in astrocytes. Furthermore, it is not known whether reactive astrocytes surviving intense oxidative stress can still support nearby neurons. The data in this Brief Report suggest that primary cortical astrocytes surviving high concentrations of the oxidative toxicant paraquat are completely resistant against subsequent oxidative challenges of the same intensity. Inhibitors of multiple endogenous defenses (e.g., glutathione, heme oxygenase 1, ERK1/2, Akt) failed to abolish or even reduce their stress resistance. Stress-reactive cortical astrocytes surviving intense oxidative stress still managed to protect primary cortical neurons against subsequent oxidative injuries in neuron/astrocyte co-cultures, even at concentrations of paraquat that otherwise led to more than 80% neuron loss. Although our previous work demonstrated a lack of stress tolerance in primary neurons exposed to dual paraquat hits, here we show that intensely stressed primary neurons can resist a second hit of hydrogen peroxide. These collective findings suggest that stress-reactive astroglia are not necessarily neurotoxic, and that severe oxidative stress does not invariably lead to stress exacerbation in either glia or neurons. Therefore, interference with the natural functions of stress-reactive astrocytes might have the unintended consequence of accelerating neurodegeneration.

## Introduction

According to the dual-hit theory of neurodegeneration, cellular exposure to severe stress—defined as stress that is lethal to some fraction of the cellular population—may render neurons more sensitive to subsequent challenges, leading to stress-induced exacerbation of cellular toxicity (Cory-Slechta et al., 2005; Carvey et al., 2006; Hawkes et al., 2007; Zhu et al., 2007; Boger et al., 2010; Gao et al., 2011). In contrast, exposure to mild (i.e., sublethal) stress can result in stress tolerance and make cells resistant against a second, more intense hit (Dirnagl et al., 2003; Eisen et al., 2004; Calabrese, 2008, 2016a,b; Leak, 2014; Thushara Vijayakuma et al., 2015). There are, however, exceptions to these distinct responses to mild versus severe stress, as we previously reported that primary cortical astrocytes surviving exposure to severe proteotoxic stress do not respond to subsequent proteotoxic challenges with additional cell loss, but acquire tolerance instead (Titler et al., 2013; Gleixner et al., 2016). Apart from proteotoxic stress, oxidative stress is another major hallmark of brain injury and disease, and it is not known whether primary astrocytes surviving severe oxidative toxicity exhibit stress tolerance, stress exacerbation, or no change in response to a second stressor. Thus, the first objective of the present study was to test the hypothesis that primary cortical astrocytes surviving severe oxidative toxicity tolerate a second oxidative hit of the same intensity with no additional cell loss.

Exposure to the herbicide paraquat is a risk factor for neurodegeneration and is commonly employed to model oxidative stress, as it inhibits complex 1 of the electron transport chain and increases superoxide levels (Bove et al., 2005; Brown et al., 2006; Cicchetti et al., 2009; Cannon and Greenamyre, 2010; Tanner et al., 2011; Tieu, 2011; Blesa et al., 2012; Caudle et al., 2012; Baltazar et al., 2014; Goldman, 2014). Aside from causing neurodegeneration, paraquat delivered *in vivo* can elicit senescence (Chinta et al., 2018), activation, and cell death in astrocytes (Shin-ichi et al., 1999; Bo et al., 2016). Activated astrocytes have been shown to aid in the recovery of brain function after injuries (Escartin and Bonvento, 2008; White and Jakeman, 2008; Sen et al., 2011; Sims and Yew, 2017), but can also be neurotoxic (Pekny and Pekna, 2014; von Bernhardi et al., 2016; Ong et al., 2017; Zorec et al., 2017). Thus, the second objective of the present study was to determine if reactive cortical astrocytes surviving paraquat exposure would subsequently injure or protect primary cortical neurons. The answer to this question has clinical implications, as pharmacological inhibition of stress-reactive astrocytes might have negative consequences on the progression of neurodegenerative disorders if reactive astrocytes continue to protect neighboring neurons under conditions of severe oxidative injury.

## Materials and Methods

Procedures were approved by the Duquesne IACUC and in accordance with the *NIH Guide for the Care and Use of Laboratory Animals*. Cortical rat pup tissue was dissected on postnatal days 1-3, and astrocytes were harvested as described (Titler et al., 2013; Gleixner et al., 2016). In order to induce oxidative stress, the first hit of paraquat (Cat. No. US-PST-740, Fisher Scientific, Hampton, NH, United States) was added to existing media on day 5 after plating. After 24 h, media was fully exchanged to remove the first paraquat hit, and cells were exposed to the second paraquat hit. Viability assays were conducted as described, 24 h after the second hit, on day 7 (Titler et al., 2013; Gleixner et al., 2016). For neuron/astrocyte bilayer co-cultures and for neuron monolayer cultures, primary cortical neurons were harvested on postnatal day 0–1 and plated on their own or on top of the astrocytes, according to previously described methods (Posimo et al., 2013, 2014, 2015). Two days after plating neurons on the layer of astrocytes, co-cultures were treated with paraquat and assayed 48h later via In-Cell Western analyses for the specific neuron markers microtubule associated protein-2 (MAP2) (1:2000 anti-MAP2; EMD Millipore, Billerica, MA, United States) and neuronal nuclei (NeuN) (1:3000 anti-NeuN; EMD Millipore).

For additional information, including rationale for the inhibitor concentrations employed, please consult the Supplementary Methods.

## Results

### Astrocytes Surviving Severe Oxidative Stress Are Invulnerable to Further Oxidative Challenges and Display Large Nuclei

**FIGURE 1:**
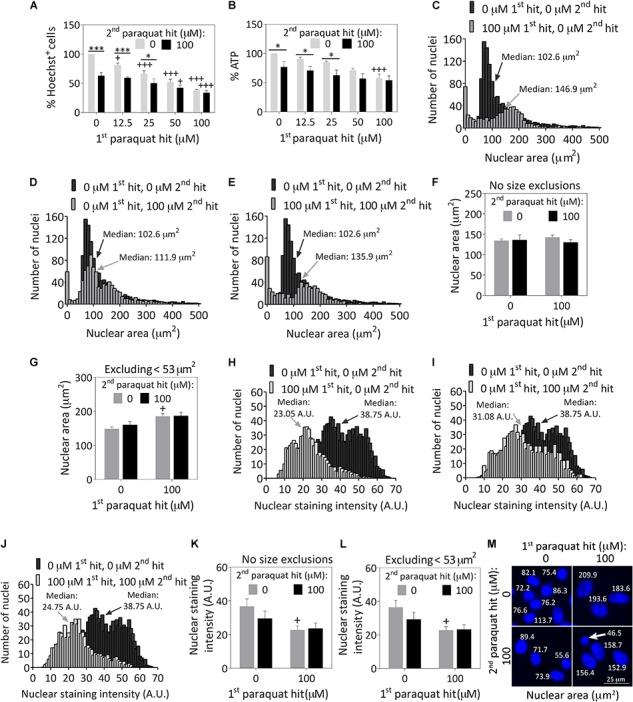
Severely stressed primary cortical astrocytes are resistant against a second oxidative insult of the same intensity and display large nuclei. Primary cortical astrocytes were treated with vehicle (phosphate buffered saline/PBS) or increasing concentrations of 12.5–100 μM paraquat on day 5, followed by a second hit of vehicle or 100 μM paraquat 24 h later. Cells were fixed and stained for the Hoechst reagent on day 7. **(A)** Blinded cell counts of Hoechst^+^ nuclei are shown. **(B)** The Cell Titer-Glo luminescence assay was used to measure ATP levels. Hoechst^+^ nuclear areas were measured on day 7 and plotted as separate frequency histograms for the groups treated with **(C)** vehicle (black histogram in background) or the first hit (gray histogram in foreground), **(D)** vehicle (black histogram in background) or the second hit (gray histogram in foreground), and **(E)** vehicle (black histogram in background) or dual hits (gray histogram in foreground). The arrows point to median areas for each distribution. **(F)** Average nuclear areas for all cells, with no size exclusions. **(G)** Average nuclear areas for all cells, excluding the small nuclei less than 53 μm^2^ in area. **(H–J)** Frequency distributions for vehicle or paraquat-treated cells as a function of Hoechst nuclear staining intensity. **(K)** Average nuclear staining intensity for all cells in each group, with no size exclusions. **(L)** Average nuclear staining intensity for all cells in each group, excluding the small cells with nuclear areas less than 53 μm^2^. **(M)** Photomontage of representative Hoechst-stained nuclei in all four groups, with indicated nuclear sizes after application of the threshold function in ImageJ. The arrow in **M** points to a cell excluded from the viability assays in other figures because its nuclear area fell below the 53 μm^2^ area threshold. Data in panels **A,B**, **F,G**, and **K,L** are shown as the mean +SEM of 3–4 independent experiments, each run in triplicate. ^+^*p* ≤ 0.05, ^+++^*p* ≤ 0.001 vs. 0 μM first paraquat hit; ^∗^*p* ≤ 0.05, ^∗∗∗^*p* ≤ 0.001 vs. 0 μM second paraquat hit; two-way ANOVA followed by the Bonferroni *post hoc* correction. Data in the frequency distributions were gathered from four independent experiments.

Astrocytes were treated with 12.5–100 μM paraquat for the first hit and 100 μM paraquat as the second hit (Figure 1A,B), based on a series of concentration-response curves (Supplementary Figures S1A–F). Cells surviving the first hits of 50 and 100 μM paraquat were resistant to a second hit of 100 μM paraquat at the structural and functional levels, based on blinded Hoechst^+^ cell counts (Figure 1A and Supplementary Figure S1G) and ATP measurements (Figure 1B).

Next, we measured the areas of all Hoechst^+^ nuclei following exposure to dual hits of 100 μM paraquat and plotted the results as frequency histograms. In the vehicle-treated control group shown in Supplementary Figure S1H, there was a small population of nuclei less than ∼50 μm^2^ in area, and a much larger distribution of cells with nuclei approximately 100 μm^2^ in median area. The first hit was toxic, as expected—it increased the population of small cells and dramatically decreased the total number of larger-sized cells (gray bars in foreground of Figure 1C) compared to the vehicle-treated control group (black bars in background of Figure 1C). Median nuclear area shifted from 100 μm^2^ to almost 150 μm^2^ after the first hit (Figure 1C). The second toxic hit by itself also reduced the total number of larger cells compared to vehicle (Figure 1D), and cells exposed to dual hits displayed a similar frequency distribution as the first-hit group (Figure 1E vs. Figure 1C).

There were no significant differences in average nuclear area across groups (Figure 1F). However, it is well established that cells that are irreparably damaged and dying by apoptosis undergo nuclear shrinkage and chromatin condensation (Eidet et al., 2014). Based on those observations and our previous work with the Hoechst and TUNEL stains in primary astrocytes, we excluded cells less than 53 μm^2^ in nuclear area (Gleixner et al., 2016), and it then became evident that the first paraquat exposure may have led to nuclear hypertrophy in the remaining cell population, or that larger cells were better able to survive the toxicant (Figure 1G). Note that viability graphs in all figures except Figure 1C–F, H–K, and Supplementary Figure S1H illustrate counts of Hoechst^+^ nuclei greater than 53 μm^2^ in area, as viability measurements are supposed to reflect live cells only. These small cells do not represent a large fraction of the total population at the time of assay, as most dead cells are detached prior to fixation and are no longer present during the Hoechst staining procedure (Figure 1C–E).

**FIGURE 2:**
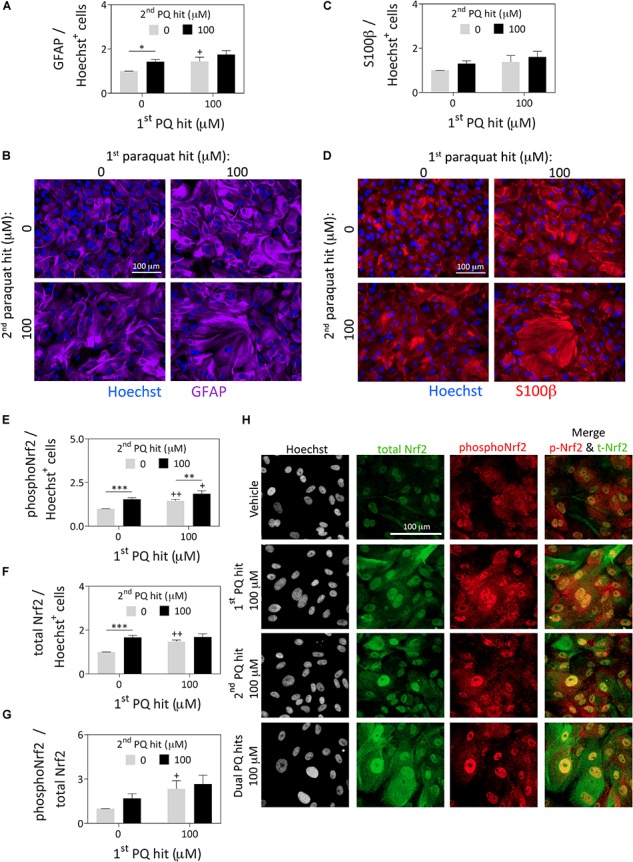
Astrocytes surviving severe paraquat toxicity display high levels of the stress-reactive marker GFAP as well as activated and total protein levels of the master transcription factor Nrf2. Primary cortical astrocytes were treated with dual hits of 100 μM paraquat or PBS as a vehicle control. One day after the second paraquat hit, astrocytes were immunostained for **(A,B)** the astrocyte marker, glial fibrillary acidic protein (GFAP; purple pseudocolor), **(C,D)** the astrocyte marker, S100β (red pseudocolor; same cells as in panel B for ease of comparison), and **(E–H)** phospho-Nrf2 (pseudocolored red) and total Nrf2 (pseudocolored green). The Hoechst reagent was used to counterstain nuclei (pseudocolored blue in panels **B** and **D** and gray in panel **H**). Protein content was measured by In-Cell Western analyses and expressed as a function of Hoechst^+^ cell numbers in the same well, to control for differences in cell densities across groups (see Supplementary Methods). All photos were captured at the same camera and software settings. Omission of primary antibodies led to loss of signal. Data in panels **A, C**, and **E–G** are shown as the mean +SEM of 5 independent experiments, each run in triplicate. ^+^*p* ≤ 0.05, ^++^*p* ≤ 0.01 vs. 0 μM first paraquat hit; ^∗^*p* ≤ 0.05, ^∗∗^*p* ≤ 0.01, ^∗∗∗^*p* ≤ 0.01 vs. 0 μM second paraquat hit; two-way ANOVA followed by the Bonferroni *post hoc* correction.

Paraquat exposure led to a slight decrease in nuclear staining intensity (Figure 1H–J), instead of the increase in chromatin staining observed during apoptosis (Hughes and Mehmet, 2003). The nuclear staining intensity was significantly lower in the first hit group with or without size exclusions (Figure 1K,L). These collective results demonstrate that paraquat may have elicited some degree of hypertrophy, a common response for stress-reactive astrocytes (Sofroniew and Vinters, 2010; Ferrer, 2017), as is evident from the images in Figure 1M and the cytoskeletal immunostaining in Figure 2 (discussed below). Alternatively, paraquat exposure may have selectively killed only those cells with relatively small nuclei.

### Astrocytes Surviving Paraquat Are Reactive in Nature and Display Higher Levels of Both Phosphorylated and Total Nrf2

In response to paraquat treatment, astrocyte cultures displayed higher levels of the stress-responsive, astrocyte-specific cytoskeletal marker glial fibrillary acidic protein (GFAP; Figure 2A,B) by In-Cell Western analyses (see Supplementary Methods). These findings are consistent with previous reports that this cell type is activated by paraquat (Li et al., 2018). However, another astrocyte marker, S100β (do Carmo Cunha et al., 2007) was not similarly affected by paraquat (Figure 2C,D). Furthermore, paraquat may have elicited cytoskeletal hypertrophy, according to the GFAP immunostaining, consistent with the nuclear enlargements quantified in Figure 1. Alternatively, large cells with high GFAP levels were perhaps more likely to survive paraquat exposure.

The first and second hits of paraquat led to higher overall levels of both phospho-Nrf2 and total Nrf2, a master transcription factor that controls cellular redox equilibrium (Vomund et al., 2017; Figure 2E–H), with even higher levels of phospho-Nrf2 prevailing after dual paraquat hits. Phosphorylation of Nrf2 at serine residue 40 is an established marker of activation and nuclear translocation of Nrf2 (Huang et al., 2002; Niture et al., 2014), consistent with its subcellular localization after paraquat treatment in Figure 2H. These findings are consistent with prior work in neural progenitor cells showing that paraquat activates the Nrf2/ARE axis (Dou et al., 2016).

Both the first and second paraquat hits increased the expression of heme oxygenase 1 (HO1; Supplementary Figure S2A), which lies downstream of Nrf2/ARE engagement (Alam et al., 1999; Loboda et al., 2016). Therefore, we employed tin protoporphyrin (SnPPIX) to significantly inhibit HO1 activity, according to previously described methods (Yoshinaga et al., 1982) (22% reduction in activity with SnPPIX compared to vehicle; two-tailed Student’s *t* test *p* = 0.0005; Gleixner and Leak, unpublished). However, there was no loss of astrocyte stress tolerance with SnPPIX, despite the basal toxicity observed with the inhibitor (Supplementary Figure S2B). Higher concentrations of SnPPIX could not be tested as they were excessively toxic in this model.

### Paraquat-Treated Astrocytes Remain Resistant to Additional Cell Loss, Despite Inhibition of Glutathione Synthesis or Stress Kinase Activation

**FIGURE 3:**
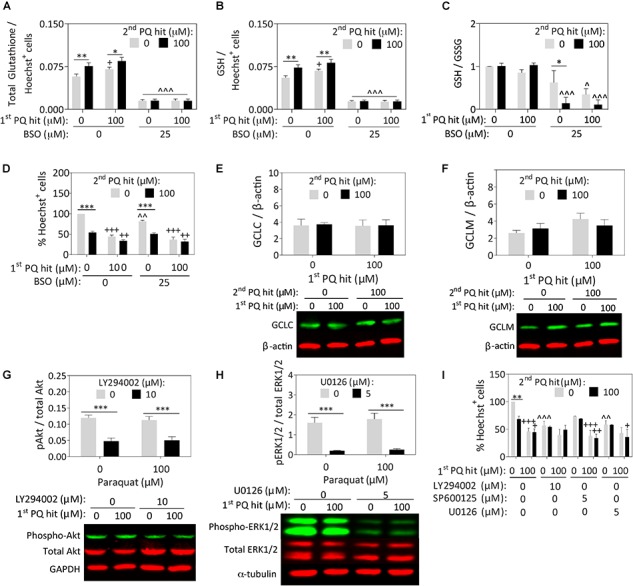
Paraquat-induced stress tolerance in astrocytes is independent of glutathione synthesis or ERK1/2 and Akt kinase activation. Primary cortical astrocytes were treated with single or dual hits of 100 μM paraquat (or equivalent volumes of PBS as a vehicle control), in the presence of pharmacological inhibitors of glutathione synthesis or stress kinases (or their respective vehicles). **(A–D)** The GSH/GSSG-Glo luminescence assay was used to measure total **(A)** and reduced **(B)** glutathione levels after treatment with an inhibitor of glutathione synthesis, buthionine sulfoximine (BSO) or its vehicle. Measurements were calculated relative to standard curves and then expressed as a function of Hoechst^+^ cell counts on a plate run in parallel. **(C)** The GSH/GSSG ratio was calculated to determine glutathione recycling. **(D)** Hoechst^+^ nuclei remaining after single or dual paraquat hits in the presence or absence of BSO were counted by a blinded observer on day 7. **(E,F)** Cell lysates were collected 24h after the second hit and the total protein levels of glutamate cysteine ligase catalytic (GCLC) and modifier (GCLM) subunits were probed by Western immunoblotting. The same loading control is shown in panels **E** and **F** because the same membrane was stained for GCLC and subsequently for GCLM (refer to Supplementary Figure S3A for full-length immunoblot). **(G,H)** Phospho-Akt was inhibited by LY294002, phospho-JNK was inhibited with SP600125, and phospho-ERK1/2 was inhibited with U0126. Lysates were collected 15 min after treatments, as phosphorylation events occur within these rapid timeframes. Phospho-JNK levels were below detection limits and are, therefore, not shown. **(I)** Blinded cell counts of astrocytes treated with dual hits of paraquat and the kinase inhibitors. Shown are the mean + SEM of 3–4 independent experiments, each run in triplicate wells, except for the Western immunoblots, which are the average of 5–7 independent experiments run in 3.5 mm dishes. For panels **A–D** and **I**, ^+^*p* ≤ 0.05, ^++^*p* ≤ 0.01, ^+++^*p* ≤ 0.001 vs. 0 μM first paraquat hit; ^∗^*p* ≤ 0.05, ^∗∗∗^*p* ≤ 0.01, ^∗∗∗^*p* ≤ 0.001 vs. 0 μM second paraquat hit; ^∧^*p* ≤ 0.05, ^∧∧^*p* ≤ 0.01, ^∧∧∧^*p* ≤ 0.001 versus 0 μM inhibitor; three-way ANOVA followed by the Bonferroni *post hoc* correction. For panels **G,H**, ^∗∗∗^*p* ≤ 0.001 vs. 0 μM inhibitor, two-way ANOVA followed by Bonferroni *post hoc* correction.

Paraquat has been reported to deplete glutathione levels in rat cortical neurons (Schmuck et al., 2002). However, Nrf2 activation typically enhances glutathione-mediated redox equilibrium (Harvey et al., 2009; Tonelli et al., 2018). In our astrocyte model, exposure to the first, second, and dual paraquat hits led to higher levels of total (Figure 3A) and reduced glutathione (GSH; Figure 3B), with no significant change in oxidized glutathione (GSSG) (Supplementary Figure S2C), or in the GSH/GSSG ratio (Figure 3C). An inhibitor of glutathione synthesis, buthionine sulfoximine (BSO), was also applied to significantly depress glutathione levels (Figure 3A–D and Supplementary Figures S2C,D). BSO successfully decreased total and reduced glutathione levels in all the groups, and reduced the GSH/GSSG ratio in all but the vehicle-treated group (Figure 3A–C). The latter result suggests that the enzyme inhibited by BSO—glutamate-cysteine ligase (previously known as gamma-glutamylcysteine synthetase)—is perhaps not activated in the vehicle group but is indeed activated after paraquat treatment. Nonetheless, significant loss of reduced glutathione levels with BSO exerted no impact on the capacity of pre-stressed astrocytes to survive the second paraquat hit (Figure 3D and Supplementary Figure S2D).

Surprisingly, we failed to observe any paraquat-induced changes in the expression of the glutamate cysteine ligase catalytic and modifier subunits (Figure 3E,F), suggesting that post-translational modifications in this protein, rather than changes in overall expression levels, may contribute to the increase in reduced glutathione levels after paraquat treatment. We did not pursue this line of inquiry further because the mechanism underlying glial stress-tolerance was not dependent on glutathione synthesis.

As paraquat increases production of the superoxide free radical (Cocheme and Murphy, 2008), we probed for the antioxidant enzyme Cu/Zn superoxide dismutase, which also lies downstream of Nrf2 activation (Zhu et al., 2005; Dreger et al., 2009), but did not observe any paraquat-induced changes in this measure (not shown).

Next, we suppressed the phosphorylation of Akt (with LY294002), ERK1/2 (with U0126), and JNK (with SP600125), as all three kinases are activated by oxidative stress and involved in stress tolerance in neuronal cells (Leak et al., 2006). Western blotting experiments revealed that the inhibitors elicited the desired effects on Akt and ERK1/2 (Figure 3G,H), but phospho-JNK levels remained below the limits of detection. Full-length immunoblots for all probings are displayed in Supplementary Figure S3. In this series of experiments, we failed to observe any change in phospho-Akt or phospho-ERK1/2 levels with paraquat, or any reduction in astrocyte stress resistance with their inhibition (Figure 3I and Supplementary Figure S2E).

### Astrocytes Are Not the Only Cell Type to Display Tolerance Against Oxidative Stress

Our previous report showed that neurons respond to dual hits of high concentrations of paraquat with additive cell loss (Heinemann et al., 2016), suggesting that neurons do not develop stress tolerance in response to severe oxidative toxicity. However, there are many other types of oxidative stimuli, including hydrogen peroxide. Therefore, we examined the response to dual hits of hydrogen peroxide in primary cortical neurons, and we discovered that the neurons surviving a first hit of 6.25 μM or higher did not respond to a second hit of 12.5 μM with significant additional cell loss according to the MAP2 In-Cell Western viability assay (Supplementary Figures S2F,G). In addition, pretreatment with 25 μM hydrogen peroxide left behind a population of MAP2^+^ neurons that did not respond to concentrations of up to 25 μM hydrogen peroxide with additional cell loss.

When we assessed functional tolerance with an assay for ATP, the first hit of hydrogen peroxide caused no loss of ATP when delivered on its own. Furthermore, the significant loss of ATP in the control group in response to the second hit of 12.5 μM hydrogen peroxide was completely prevented with pre-exposure to first hits of 12.5 μM or higher (Supplementary Figure S2H). The highest concentration of the first hit (25 μM) also prevented ATP loss in response to a second hit of 25 μM hydrogen peroxide.

These structural and functional data reveal that glia are not the only cells that display tolerance against intense oxidative stress. Rather, there appears to be a dramatic upregulation of ATP in the neurons that manage to survive exposure to severe oxidative stress and a preconditioning-like effect of hydrogen peroxide on their metabolic viability.

### Paraquat-Stressed Astrocytes Continue to Fulfill Their Neuroprotective Roles, Despite Potential Cellular Hypertrophy, Increased Expression of the Stress-Reactive Marker GFAP, and a Significant Reduction in Cell Numbers

The second main objective of the present study was to determine if reactive cortical astrocytes surviving paraquat exposure would subsequently injure or protect primary cortical neurons, or elicit no change in neuronal survival. Previous studies have already shown that astrocytes can protect neurons from rotenone and paraquat toxicity (Rathinam et al., 2012), but it is not known if they can continue to support neurons even after they survive high concentrations of paraquat that are lethal to a fraction of the cells.

**FIGURE 4:**
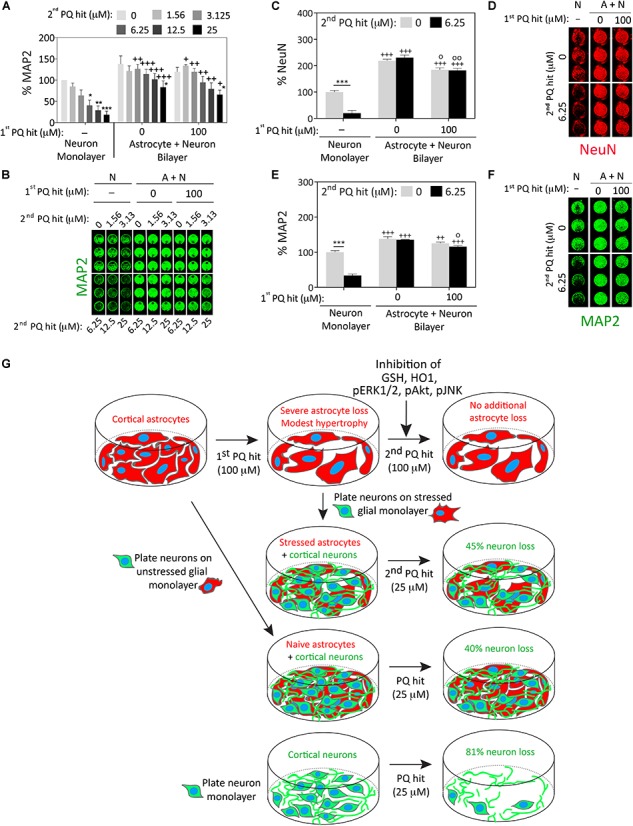
Astrocytes continue to fulfill their neuroprotective roles under conditions of severe oxidative toxicity. Primary cortical neurons were plated as monolayers or over paraquat-stressed or vehicle-treated (unstressed) primary cortical astrocytes and treated 2 days later with the indicated concentrations of paraquat (defined as the second hit; this is the first hit for the neurons and the second hit for the astrocytes). Cells were fixed after two days and neuron viability was assessed by In-Cell Western analyses for the specific neuron markers microtubule associated protein 2 (MAP2; panels **A,B** and **E,F**) or Neuronal Nuclei (NeuN; **C,D**). **(G)** Summary schematic. Data in panels **A–F** are shown as the mean + SEM of 3–4 independent experiments. For data in panels **A–E**, ^∗^*p* ≤ 0.05, ^∗∗^*p* ≤ 0.01, ^∗∗∗^*p* ≤ 0.001 vs. 0 μM second paraquat hit; ^+^*p* ≤ 0.05, ^++^*p* ≤ 0.01, ^+++^*p* ≤ 0.001 vs. neuron monolayer cultures; °*p* ≤ 0.05, °°*p* ≤ 0.01 vs. 0 μM first paraquat hit in neuron + astrocyte (A+N) bilayer cultures; three-way ANOVA followed by the Bonferroni *post hoc* correction.

There was a significant improvement in neuronal viability when neurons cultured over previously stress-naïve astrocytes were exposed to the second paraquat hit (3.125 μM and higher), compared to neuron monolayers (Figure 4A,B). Furthermore, astrocytes surviving the first paraquat hit continued to robustly protect primary cortical neurons plated on top of the glial monolayer, even against the highest concentration of the second hit (25 μM; Figure 4A,B). The concentrations of paraquat were lower for the second hit than the first hit because the neurons in the control group (neuron cultures without an astrocyte layer beneath them) were far more vulnerable to paraquat than astrocytes, consistent with previous studies (Schmuck et al., 2002).

We confirmed these findings by repeating the co-culture studies with two independent markers for neurons (MAP2 and NeuN), employing 100 μM paraquat as the first hit in astrocyte cultures and 6.25 μM paraquat as the second hit in the glia/neuron co-cultures (Figure 4C–F). In this series of experiments, astrocytes surviving the first paraquat hit were only marginally less neuroprotective than naïve, unstressed astrocytes.

These collective results suggest that astrocytes surviving high concentrations of oxidative toxicity can still protect nearby neurons from intense paraquat toxicity, despite exposure to dual hits of severe stress and a significant reduction in glial cell numbers (see summary in Figure 4G).

## Discussion

In the present study, we examined oxidative stress tolerance in primary cortical astrocytes exposed to dual hits of high concentrations of the xenobiotic paraquat. Tolerance against severe oxidative stress was evident at both the structural and functional levels, according to the Hoechst and ATP viability assays, respectively. There are at least two explanations for the glial stress tolerance observed here: 1) The first paraquat hit upregulates unknown defenses that temper the toxicity of subsequent paraquat challenges in the surviving astrocytes, or 2) paraquat exposure leaves behind only those cells with *constitutively* higher expression of defensive molecules, but there is no further active upregulation of natural defenses. For example, there might be selection for cells that have naturally high levels of Nrf2, after treatment with paraquat, as Nrf2 has been linked to increased resistance against oxidative stress (Ma, 2013). In this latter scenario, paraquat kills only the most vulnerable cells (expressing low levels of Nrf2 and its downstream proteins), and the surviving cultures are then enriched in the most paraquat-resistant members of the original population. Although there was a hormetic increase in reduced glutathione levels after paraquat exposure, this was not responsible for glial stress tolerance in the current model. However, there are other molecules downstream of Nrf2 activation, besides glutathione, HO1, and superoxide dismutase, that might mediate glial stress resistance.

In our previous work with dual hits of proteasome inhibitors, we ruled out the second of the abovementioned two explanations, because the dual proteotoxic hits elicited synergistic upregulation of HO1 and ubiquitinated proteins (compared to single hits), suggesting that the cells remaining alive after the first hit continued to *actively* respond to the second hit (Titler et al., 2013). Furthermore, glutathione depletion in those studies unmasked the toxic effects of the second proteotoxic hit, demonstrating that cells surviving the first hit were indeed vulnerable to the second hit (provided actively engaged defense systems were inhibited) and did not display naturally higher baseline defenses from the beginning (Titler et al., 2013; Gleixner et al., 2016). In the present study, however, we cannot conclude with certitude that the cells surviving the first paraquat hit *actively* respond to the second hit. In either case, our data reveal heterogeneity in the cortical astrocyte population under study, and it is possible that this reflects the existence of neurotoxic A1 versus neuroprotective A2 astrocyte phenotypes (Filous and Silver, 2016; Liddelow et al., 2017). In our model, an extensive morphometric analysis suggested that the first paraquat hit may lead to astrocyte hypertrophy, a common response in injured glia (Sofroniew and Vinters, 2010; Chinta et al., 2018). In contrast, astrocytes shrink in size after exposure to A1 phenotype-inducing stimuli (Liddelow et al., 2017). Although we employed A1 and A2 markers (not shown), they were not specific in our hands, and we cannot conclude that the surviving astrocytes conform to A2 criteria based on an increase in size alone. Further studies to investigate the phenotypic polarization of the paraquat-resistant population are worth considering.

Aside from A1/A2 phenotypes, astrocytes also display inter- and intraregional heterogeneity depending upon the brain region that they populate (Farmer and Murai, 2017). Although we limited our dissections to the cerebral cortex, the cortex is a relatively large brain region with a columnar organization, six cell layers, four lobes, and numerous lobar subregions with distinct functions. Therefore, it is possible that we captured the distinct responses of heterogenous astrocytes within the cortical edifice of the telencephalon.

The second major finding of the present study is that astrocytes exposed to severe oxidative injury can still robustly protect neighboring neurons from intense oxidative toxicity, although they are significantly reduced in numbers and activated by paraquat. The difference in neuroprotective potential between previously stressed and stress-naïve astrocytes was unexpectedly minor, which is consistent with the view that astrocytes must protect surrounding cells under both physiological and pathological conditions—even after they have been exposed to intense stress that injures and kills some of their neighbors. For example, in spinal cord injury, reactive astrocytes can mitigate tissue loss and motor dysfunction (Faulkner et al., 2004). Although glial scars are known to inhibit axonal growth, they also form a physical barrier between injured tissue and surrounding cells, which mitigates the spread of neuroinflammatory secretions and deescalates a wave of secondary degeneration (White and Jakeman, 2008; Rolls et al., 2009). In this study, the neurosupportive capacities of stressed astrocytes were evident even at the highest concentration of paraquat tested, which elicited >80% loss of neuronal viability in neuron monolayers but only 45% loss of neuronal viability in stressed glia/neuron bilayers (see summary in Figure 4G). Therefore, our findings are consistent with the view that reactive astrocytes continue to scavenge toxic molecules such as free radicals and excess ions, and provide trophic support and metabolic nutrients to surrounding cells.

Although many have argued that reactive astrocytes aid in the recovery of function after brain injuries (Escartin and Bonvento, 2008; White and Jakeman, 2008; Sen et al., 2011; Sims and Yew, 2017), reactive astrocytes can also assume toxic roles (Pekny and Pekna, 2014; von Bernhardi et al., 2016; Ong et al., 2017; Zorec et al., 2017). Stress-reactive astrocytes are viewed as a double-edged sword in the injured brain (Cabezas et al., 2013), and many studies emphasize their neurotoxicity (Sidoryk-Wegrzynowicz et al., 2011; Liddelow et al., 2017). For example, reactive glia may drive abnormal neuronal activity and synaptic dysfunction (Chung et al., 2015; Robel and Sontheimer, 2016), and conditioned media from paraquat-stressed senescent human astrocytes may induce dopaminergic neurodegeneration (Chinta et al., 2018). Exposure of U373 cells to sublethal levels of paraquat for seven days reduced their ability to protect SH-SY5Y cells, perhaps by impacting autophagic capacity (Janda et al., 2015). On the other hand, reactive glia have also been shown to protect dopaminergic neurons from MPTP and 6-hydroxydopamine toxicity (Jakel et al., 2007; Chen et al., 2009; Gardaneh et al., 2011). Furthermore, exposure of astrocytes to sublethal oxidative stress contributes to neuroprotection in models of ischemia and glutathione depletion (Haskew-Layton et al., 2010; Bell et al., 2011), and primary astrocytes have been shown to protect neurons from paraquat, rotenone, and ethanol toxicity (Watts et al., 2005; Mullett and Hinkle, 2009, 2011; Rathinam et al., 2012; Mullett et al., 2013). It is important to note that the latter studies did not determine if *severely* stressed primary astrocytes still display neuroprotective properties as we did. In addition, we have demonstrated that the fraction of astrocytes that is able to survive high concentrations of oxidative stress protects neurons almost as well as untreated astrocytes in astrocyte/neuron co-cultures.

One limitation of the current investigation is that astrocytes were harvested from young animals and examined in isolation from other cell types, such as pro-inflammatory microglia. However, in an *in vivo* study we would not have been able to expose only astrocytes (and not any other cell types) to paraquat toxicity to specifically determine whether astrocyte responses to oxidative stress blunt the toxicity of subsequent neuronal injuries. A second limitation is that the pharmacological inhibitors may have off-target effects. On the other hand, if the inhibitors exerted off-target effects in our model, it seems all the more impressive that the stress tolerance was completely unaffected by their application. Third, some of the inhibitors may not have been applied at sufficiently high concentrations. For example, the degree of inhibition of HO1 activity in primary astrocytes with SnPPIX was only 22%. However, we chose our inhibitor concentrations based on the literature and pilot studies (see Supplementary Methods) and found that exposure to higher concentrations were not practical, because they killed most of the astrocytes by the time of assay.

## Conclusion

Considered together with previous work (Titler et al., 2013; Gleixner et al., 2016), the present findings reveal that astrocyte stress resistance is generalizable across different classes of acute insults, and that astrocytes exposed to various types of stress do not lose their neuroprotective functions even when the stress is severe. Thus, astrocytes may have evolved to tolerate severe oxidative stress in order to continue to fulfill their supportive roles in highly damaged brains. However, oxidative stress tolerance was not unique to astrocytes, as we also found that neurons failed to respond to dual hits of hydrogen peroxide with any additional cell loss and were metabolically preconditioned. These findings have important implications for neurodegenerative conditions, as they suggest that neuron loss after severe oxidative stress would progress at an accelerated rate were it not for the stress resistance of reactive glia and neurons.

## Author Contributions

DP, TB, EE, RG, TD, DH, and AG conducted the experiments, analyzed the data, and generated the figures. RL designed the experiments. RL and TB wrote the manuscript. DP wrote sections of an early draft of the manuscript. AG provided editing feedback.

## Conflict of Interest Statement

The authors declare that the research was conducted in the absence of any commercial or financial relationships that could be construed as a potential conflict of interest.
