# Supplementary material for: Astrocytes Do Not Forfeit Their Neuroprotective Roles After Surviving Intense Oxidative Stress
Source: Front Mol Neurosci. 2019 Apr 5;12:87. doi: 10.3389/fnmol.2019.00087 (PMC6460290; doi:10.3389/fnmol.2019.00087)
Supplement: Supplementary file 1 [file Data_Sheet_1.docx]

**Supplementary Material**

**Astrocytes Do Not Forfeit Their Neuroprotective Roles**

**After Surviving Intense Oxidative Stress**

Tarun N. Bhatia^+^, Deepti B. Pant^+^, Elizabeth A. Eckhoff, Rachel N. Gongaware,

Timothy Do, Daniel F. Hutchison, Amanda M. Gleixner, and Rehana K. Leak*

Graduate School of Pharmaceutical Sciences, Duquesne University, Pittsburgh PA

^+^ contributed equally

Number of words in supplementary material: 1208

Number of supplementary figures: 3

Rehana K. Leak, Ph.D. * to whom correspondence should be addressed

407 Mellon Hall

600 Forbes Ave

Duquesne University

Pittsburgh PA 15282

FAX: 412.396.4660

TEL: 412.396.4734

E-mail: [leakr@duq.edu](mailto:leakr@duq.edu)

**Supplementary Methods**

1. **Pharmacological inhibitors.** Glutathione synthesis was inhibited with 25 μM buthionine sulfoximine (Cat. no. 309475000, Acros Organics, Fair Lawn, NJ), based on previous reports that this concentration depletes glutathione in astrocytes [1; 2]. Our previous work in primary cortical astrocytes confirmed that concentrations of 12.5–50 μM of BSO significantly decreased glutathione levels [3]. Based on our previous preconditioning work in dopaminergic cells [4], ERK1/2 phosphorylation was inhibited with the MEK1/2 inhibitor U0126 (5 μM; Cat. no. 1144, ToCris Bioscience, Bristol, UK), JNK activity was inhibited with SP600125 (5 μM; Cat. no. 1496, ToCris), and Akt phosphorylation was suppressed with the PI3K inhibitor LY294002 HCl (10 μM; Cat. no. 1130, ToCris). Other groups have also applied 10 μM LY294002 to inhibit PI3K/Akt in astrocytes [5; 6; 7] and SP60025 has been applied at 5 μM to inhibit JNK in cell lines [8]. Although SP60025 has been used at 10 μM concentrations in astrocytes [9], this concentration was too toxic for the present model. U0126 has previously been applied at 5 μM in primary astrocytes, hippocampal neurons, and human brain endothelial cells to inhibit phosphorylation of ERK1/2 [10; 11; 12], and higher concentrations were too toxic in the present model. HO1 activity was inhibited in the current model by 22% with tin protoporphyrin (SnPPIX; Cat. no. Sn749-9, Frontier Scientific, Logan, Utah) relative to the vehicle control (two-tailed Student’s *t* test p value = 0.0005), according to a previously described activity assay [13] (Gleixner and Leak, unpublished). Previous studies have also shown that SnPPIX inhibits heme oxygenase activity at 3.3 μM [14]. Although previous studies on astrocytic cells have applied SnPPIX at 30 μM [15], a subsequent study showed no additional effects beyond 10 μM in SH-SY5Y cells [16], and concentrations higher than 20 μM were toxic in the present model. All inhibitors were administered concurrently with paraquat on day 5 and day 6 after plating astrocytes. Viability was assayed 24h later, as described below.
2. **Cell viability assays and immunostaining.** Astrocytes were fixed in 2% paraformaldehyde and 2% sucrose in 0.1 M phosphate buffer for 20 min. Fixation was followed by three washes with 10 mM freshly filtered phosphate-buffered saline (PBS). Nuclei were then stained with the Hoechst reagent (bizBenzidine, 1:2000, cat.no. DR05500, Biostatus, UK) for 20 min. Following three additional washes with PBS, images were captured with an epifluorescent microsope with a 20× objective (EVOS Model #AMF-4301-US, Advanced Microscopy Group, Bothell, WA or Olympus IX73, B&B Microscopes, Pittsburgh, PA). Cell viability was quantified in a blinded fashion using the same threshold values across wells in ImageJ software (NIH Image, Bethesda, MD), as described previously [17], or in cellSens software (Olympus, Pittsburgh, PA). Based on our previous work with the Hoechst and TUNEL stains, cells with condensed nuclei smaller than 53 μm^2^ in surface area (350 pixels in ImageJ) were excluded from the cell counts, as viability counts are supposed to reflect live cells only [17].

Cultures were treated with dual hits of paraquat and immunostained 24h after the second hit with antibodies against the astrocyte markers, glial fibrillary acidic protein (1:1000 anti-GFAP; Dako, Glostrup, Denmark) and S100β (1:1000; Sigma-Aldrich), and the transcription factor nuclear factor (erythroid-derived 2)-like 2 (1:200 anti-phospho-Nrf2; Bioss Antibodies, Boston, MA; 1:300 anti-total-Nrf2; Novus Biologicals, Centennial, CO), followed by simultaneous exposure to infrared fluorescent secondary antibodies for In-Cell Western analyses and visible-range secondary antibodies for higher-resolution standard epifluorescent microscopy (Jackson Immunoresearch Laboratories Inc., West Grove, PA), as described previously [17; 18; 19]. Omission of primary antibodies led to loss of signal in every immunocytochemical run. Images from all groups were captured at the same camera and software settings (Olympus IX73, B&B Microscopes, Pittsburgh, PA).

For data in **Fig.S2F-H**, primary cortical neurons were cultured as described previously [18; 19], and treated on day 2 *in vitro* with the first hit of hydrogen peroxide, followed by a second hit of hydrogen peroxide on day 5. Viability assays were conducted on day 7 *in vitro*. Neuron viability was measured by the In-Cell Western technique, as described previously [18; 19; 20]. Fluorescent signal in this assay shows a strong linear correlation with neuronal numbers [18; 19].

Total, reduced (GSH), and oxidized (GSSG) glutathione levels were measured by the GSH/GSSG-Glo luminescence assay (Cat. no. V6611, Promega, Madison, WI), as per manufacturer’s instructions. Briefly, cells were incubated with glutathione lysis reagents on a plate shaker at room temperature for five minutes, after which cells were incubated in the luciferin generation reagent for 30 minutes. Finally, the luciferin detection reagent was added for 15 minutes and luminescence was assessed on a luminometer (SpectraMax, Molecular Devices, San Jose, CA). Levels of glutathione were calculated based on standard curves run in parallel, and then expressed as a fraction of Hoechst^+^ counts from parallel plates treated side-by-side with the original plate designated for the luminescence assay, because the GSH/GSSG-Glo assay involves complete cell lysis.

In order to measure cell viability at the functional level, the Cell-Titer Glo luminescent assay (Cat. no. G7572, Promega, Madison, WI) was used to detect ATP levels, as described previously [19]. Briefly, cells were incubated with the Cell-Titer Glo reagents for 10 minutes and luminescence was assessed on a luminometer (VICTOR3 1420 multilabel counter, PerkinElmer, Waltham, MA).

1. **Immunoblotting.** Cell lysates were collected 15 min or 24h following treatment, as specified in the figure legends. Cells were harvested with cell lysis buffer (recipe from Cell Signaling Technology Cat. no. 9803, Danvers, MA) containing 10 mM sodium fluoride and 1% protease inhibitor cocktail (Cat. no. P8340, Sigma-Aldrich). Equal amounts of protein were separated with standard gel electrophoresis and transferred onto nitrocellulose membranes (EMD Millipore). Membranes were washed three times with Tris-buffered saline (TBS), and incubated in 5% nonfat dry milk in TBS or a 50% Odyssey Block solution in TBS (Cat. no. 927-40000, LI-COR, Lincoln, NE). Antibody dilutions were prepared in a 50% Odyssey block solution with TBS and 0.1% Tween. All membranes were incubated with primary antibodies overnight at 4°C. Primary antibodies were employed against the phosphorylated and total kinases ERK1/2, Akt, and JNK (1:1000; Cell Signaling Technology), superoxide dismutase 1 (1:2000 anti-SOD1; Sigma-Aldrich), heme oxygenase 1 (1:300 anti-HO1; Sigma-Aldrich), glutathione cysteine ligase modifier subunit (1:1000 anti-GCLM; Sigma-Aldrich), glutathione cysteine ligase catalytic subunit (1:1000 anti-GCLC; Sigma-Aldrich), the loading control β-actin (1:50,000; Sigma-Aldrich), the loading control alpha-tubulin (1:200,000; Sigma-Aldrich), and the loading control GAPDH (1:5000; Cell Signaling Technology). Secondary antibodies (Jackson Immunoresearch Laboratories) were diluted to 1:20,000 in the same blocking solution and applied to membranes for 1 h at room temperature on the following day. Following multiple washes in TBS and 0.1% Tween, immunostained membranes were then scanned on an Odyssey infrared imager (model number 9201-01, LI-COR). Protein levels were quantified using ImageStudio software (LI-COR).
2. **Statistics**. Each viability experiment was conducted in triplicate wells in 96-well plates on at least three independent occasions, for an experimental ‘n’ of 3. Western blotting experiments were conducted on 5-7 independent occasions in one 3.5 mm dish per group. Statistical analyses were performed using IBM SPSS Statistics 20 (Armonk, NY) for the ANOVAs or GraphPad Prism for the histograms (Version 6.0, La Jolla, CA). A one, two, or three-way ANOVA was employed, always followed by the Bonferroni *post hoc* correction. Differences between groups were deemed statistically significant only when p ≤ 0.05.

**References**

[1] D.M. Pizzurro, K. Dao, and L.G. Costa, Astrocytes protect against diazinon- and diazoxon-induced inhibition of neurite outgrowth by regulating neuronal glutathione. Toxicology 318 (2014) 59-68.

[2] M.A. Brito, A.I. Rosa, A.S. Falcao, A. Fernandes, R.F. Silva, D.A. Butterfield, and D. Brites, Unconjugated bilirubin differentially affects the redox status of neuronal and astroglial cells. Neurobiol Dis 29 (2008) 30-40.

[3] A.M. Titler, J.M. Posimo, and R.K. Leak, Astrocyte plasticity revealed by adaptations to severe proteotoxic stress. Cell and tissue research 352 (2013) 427-43.

[4] R.K. Leak, A.K. Liou, and M.J. Zigmond, Effect of sublethal 6-hydroxydopamine on the response to subsequent oxidative stress in dopaminergic cells: evidence for preconditioning. J Neurochem 99 (2006) 1151-63.

[5] A.R. Nookala, A. Shah, R.J. Noel, and A. Kumar, HIV-1 Tat-mediated induction of CCL5 in astrocytes involves NF-kappaB, AP-1, C/EBPalpha and C/EBPgamma transcription factors and JAK, PI3K/Akt and p38 MAPK signaling pathways. PLoS One 8 (2013) e78855.

[6] Q. Gao, Y. Li, and M. Chopp, Bone marrow stromal cells increase astrocyte survival via upregulation of phosphoinositide 3-kinase/threonine protein kinase and mitogen-activated protein kinase kinase/extracellular signal-regulated kinase pathways and stimulate astrocyte trophic factor gene expression after anaerobic insult. Neuroscience 136 (2005) 123-34.

[7] C. Park, S. Lee, I.H. Cho, H.K. Lee, D. Kim, S.Y. Choi, S.B. Oh, K. Park, J.S. Kim, and S.J. Lee, TLR3-mediated signal induces proinflammatory cytokine and chemokine gene expression in astrocytes: differential signaling mechanisms of TLR3-induced IP-10 and IL-8 gene expression. Glia 53 (2006) 248-56.

[8] S. Dai, L. Jiang, G. Wang, X. Zhou, X. Wei, H. Cheng, Z. Wu, and D. Wei, HSP70 interacts with TRAF2 and differentially regulates TNFalpha signalling in human colon cancer cells. J Cell Mol Med 14 (2010) 710-25.

[9] T. Huang, J. Solano, D. He, M. Loutfi, W.D. Dietrich, and J.W. Kuluz, Traumatic injury activates MAP kinases in astrocytes: mechanisms of hypothermia and hyperthermia. J Neurotrauma 26 (2009) 1535-45.

[10] A. Schneider, T. Mehmood, S. Pannetier, and A. Hanauer, Altered ERK/MAPK signaling in the hippocampus of the mrsk2_KO mouse model of Coffin-Lowry syndrome. J Neurochem 119 (2011) 447-59.

[11] S. Moidunny, M. Matos, E. Wesseling, S. Banerjee, D.J. Volsky, R.A. Cunha, P. Agostinho, H.W. Boddeke, and S. Roy, Oncostatin M promotes excitotoxicity by inhibiting glutamate uptake in astrocytes: implications in HIV-associated neurotoxicity. J Neuroinflammation 13 (2016) 144.

[12] X.P. Yang, J.Y. Fu, R.C. Yang, W.T. Liu, T. Zhang, B. Yang, L. Miao, B.B. Dou, C. Tan, H.C. Chen, and X.R. Wang, EGFR transactivation contributes to neuroinflammation in Streptococcus suis meningitis. J Neuroinflammation 13 (2016) 274.

[13] T. Yoshinaga, S. Sassa, and A. Kappas, Purification and properties of bovine spleen heme oxygenase. Amino acid composition and sites of action of inhibitors of heme oxidation. J Biol Chem 257 (1982) 7778-85.

[14] H.L. Bonkovsky, J.F. Healey, and J. Pohl, Purification and characterization of heme oxygenase from chick liver. Comparison of the avian and mammalian enzymes. Eur J Biochem 189 (1990) 155-66.

[15] R.F. Regan, Y. Guo, and N. Kumar, Heme oxygenase-1 induction protects murine cortical astrocytes from hemoglobin toxicity. Neurosci Lett 282 (2000) 1-4.

[16] L. Goldstein, Z.P. Teng, E. Zeserson, M. Patel, and R.F. Regan, Hemin induces an iron-dependent, oxidative injury to human neuron-like cells. J Neurosci Res 73 (2003) 113-21.

[17] A.M. Gleixner, J.M. Posimo, D.B. Pant, M.P. Henderson, and R.K. Leak, Astrocytes Surviving Severe Stress Can Still Protect Neighboring Neurons from Proteotoxic Injury. Mol Neurobiol 53 (2016) 4939-60.

[18] J.M. Posimo, A.M. Titler, H.J. Choi, A.S. Unnithan, and R.K. Leak, Neocortex and allocortex respond differentially to cellular stress in vitro and aging in vivo. PLoS One 8 (2013) e58596.

[19] J.M. Posimo, A.S. Unnithan, A.M. Gleixner, H.J. Choi, Y. Jiang, S.H. Pulugulla, and R.K. Leak, Viability assays for cells in culture. Journal of visualized experiments : JoVE 83 (2014) e50645.

[20] J.M. Posimo, J.N. Weilnau, A.M. Gleixner, M.T. Broeren, N.L. Weiland, J.L. Brodsky, P. Wipf, and R.K. Leak, Heat shock protein defenses in the neocortex and allocortex of the telencephalon. Neurobiol Aging 36 (2015) 1924-37.
