# Supplementary material for: Astrocytes Do Not Forfeit Their Neuroprotective Roles After Surviving Intense Oxidative Stress
Source: Front Mol Neurosci. 2019 Apr 5;12:87. doi: 10.3389/fnmol.2019.00087 (PMC6460290; doi:10.3389/fnmol.2019.00087)
Supplement: Supplementary file 2 [file Data_Sheet_2.pdf]

Figure S1

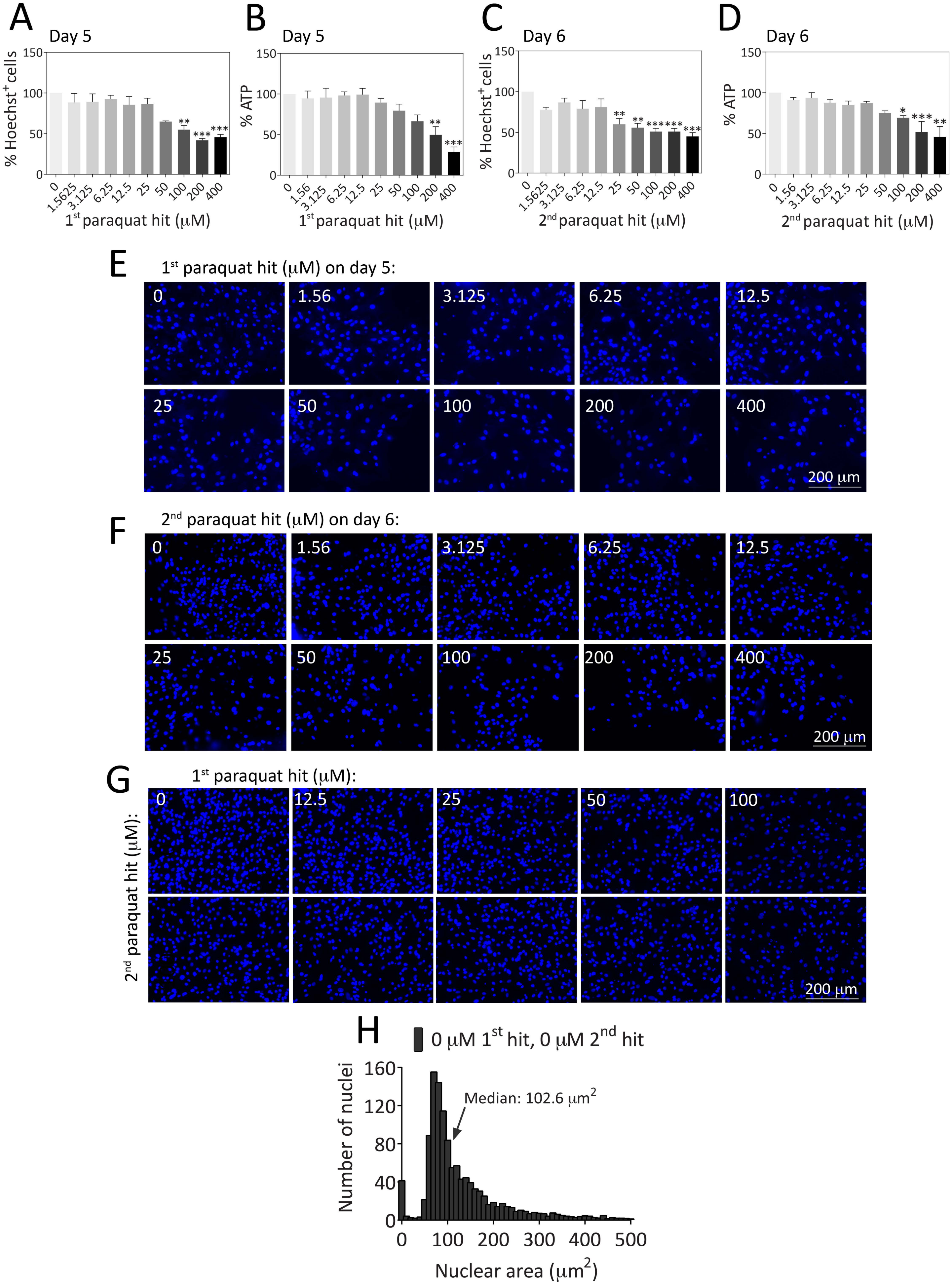
A
Day 5
% Hoechst+ cells
1st paraquat hit (μM)
B
Day 5
% ATP
1st paraquat hit (μM)
C
Day 6
% Hoechst+ cells
2nd paraquat hit (μM)
D
Day 6
% ATP
2nd paraquat hit (μM)
0
1.5625
1.56
3.125
6.25
12.5
25
50
100
200
400
150
E
1st paraquat hit (μM) on day 5:
200 μm
F
2nd paraquat hit (μM) on day 6:
200 μm
G
1st paraquat hit (μM):
2nd paraquat hit (μM):
200 μm
H
0 μM 1st hit, 0 μM 2nd hit
Number of nuclei
Median: 102.6 μm2
Nuclear area (μm2)
160
120
80
40
300
500

Figure S1. Primary cortical astrocytes are sufficiently stressed by paraquat to display significant loss of structural and metabolic viability. Primary cortical astrocytes were treated with the indicated concentrations of paraquat or PBS. (**A-B**) Treatments were administered 5 days after plating and viability was assessed 48h following treatment (on day 7) by counting Hoechst-stained nuclei and measuring ATP levels. (**C-D**) Paraquat was administered 6 days after plating, and viability assays were performed 24h later. (**E**) Representative images of Hoechst-stained astrocytic nuclei for the data shown in panel A. (**F**) Representative images of Hoechst^{+} astrocytic nuclei for the data shown in panel C. (**G**) Representative images of Hoechst-stained nuclei fixed on day 7 following the first and second paraquat hits, corresponding to the blinded counts in **Figure 1A**. (**H**) Frequency histogram for nuclear areas of vehicle-treated cells. Data in panels A-D are shown as the mean + SEM of 3-4 independent experiments, each run in triplicate. For panels A-D: * $p \leq 0.05$, ** $p \leq 0.01$, *** $p \leq 0.001$ vs 0 μM paraquat; one-way ANOVA followed by the Bonferroni *post hoc* correction. Data in the frequency distribution were gathered from 4 independent experiments.

Figure S2

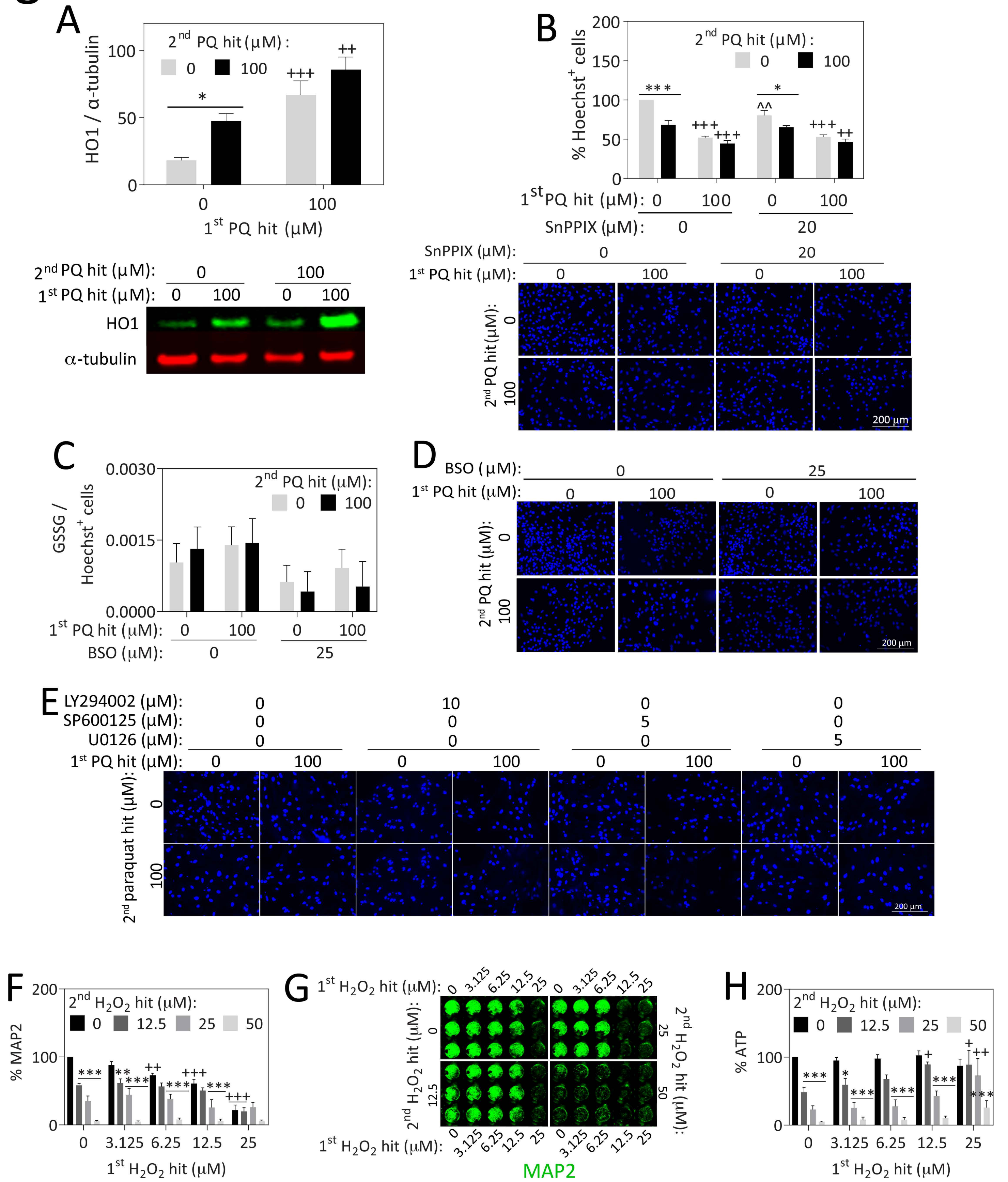

A
HO1 / α-tubulin
2nd PQ hit (μM):
0
100
1st PQ hit (μM)
B
% Hoechst+ cells
SnPPIX (μM):
HO1
α-tubulin
200 μm
C
GSSG / Hoechst+ cells
BSO (μM):
D
E
LY294002 (μM):
SP600125 (μM):
U0126 (μM):
2nd paraquat hit (μM):
F
% MAP2
2nd H2O2 hit (μM):
1st H2O2 hit (μM)
G
MAP2
H
% ATP

Figure S2. Primary cortical astrocytes do not lose stress resistance when heme oxygenase 1 activity is inhibited, and stressed primary cortical neurons can tolerate some concentrations of hydrogen peroxide. (**A**) Astrocyte cell lysates were collected 24h after the second paraquat hit, and heme oxygenase 1 (HO1) levels were probed by Western immunoblotting. (**B**) Tin protoporphyrin (SnPPIX) was used to inhibit HO1, and Hoechst$^+$ nuclei were counted by a blinded observer on day 7 (24h after the second hit). (**C**) Primary cortical astrocytes were treated with dual hits of paraquat and with buthionine sulfoximine (BSO) to inhibit glutathione synthesis. The GSH/GSSG-Glo luminescence assay was used to assess oxidized glutathione levels on day 7 (24h after the second hit), relative to a standard curve, and expressed as a fraction of cell counts. (**D**) Representative Hoechst$^+$ nuclei following exposure to paraquat and BSO, corresponding to data in **Figure 3D**. (**E**) Representative Hoechst$^+$ nuclei following exposure to paraquat and the inhibitors of multiple stress-responsive kinases, LY294002 (inhibits phospho-Akt via PI3K suppression), U0126 (inhibits phospho-ERK1/2 via MEK1/2 suppression), and SP600125 (inhibits phosphor-JNK), corresponding to data in **Figure 3I**. (**F**) Primary cortical neurons were treated with the first hit of hydrogen peroxide on day 2 and the second hit of hydrogen peroxide on day 5. Cells were fixed on day 7 and assessed for neuronal viability with the In-Cell Western assay for MAP2. (**G**) Representative In-Cell Western for the data shown in panel F. (**H**) Primary cortical neurons treated with dual hits of hydrogen peroxide were assessed for ATP levels by the Cell Titer-Glo luminescence assay on day 7. Shown are the mean + SEM of at least 3 independent experiments, each run in triplicate wells, except for the Western immunoblots, which are the average of 5-7 independent experiments run in single 3.5 mm dishes. For panels A, F, H, $^{+}$ $p \leq 0.05$, $^{++}$ $p \leq 0.01$, $^{+++}$ $p \leq 0.001$ vs 0 μM first toxic hit; * $p \leq 0.05$, ** $p \leq 0.01$ *** $p \leq 0.001$ vs 0 μM second toxic hit; two-way ANOVA followed by the Bonferroni *post hoc* correction. For panel B, $^{++}$ $p \leq 0.01$, $^{+++}$ $p \leq 0.001$ vs 0 μM first paraquat hit; * $p \leq 0.05$, *** $p \leq 0.001$ vs 0 μM second paraquat hit; $^{\wedge\wedge}$ $p \leq 0.01$ versus 0 μM SnPPIX; three-way ANOVA followed by the Bonferroni *post hoc* correction.

Figure S3

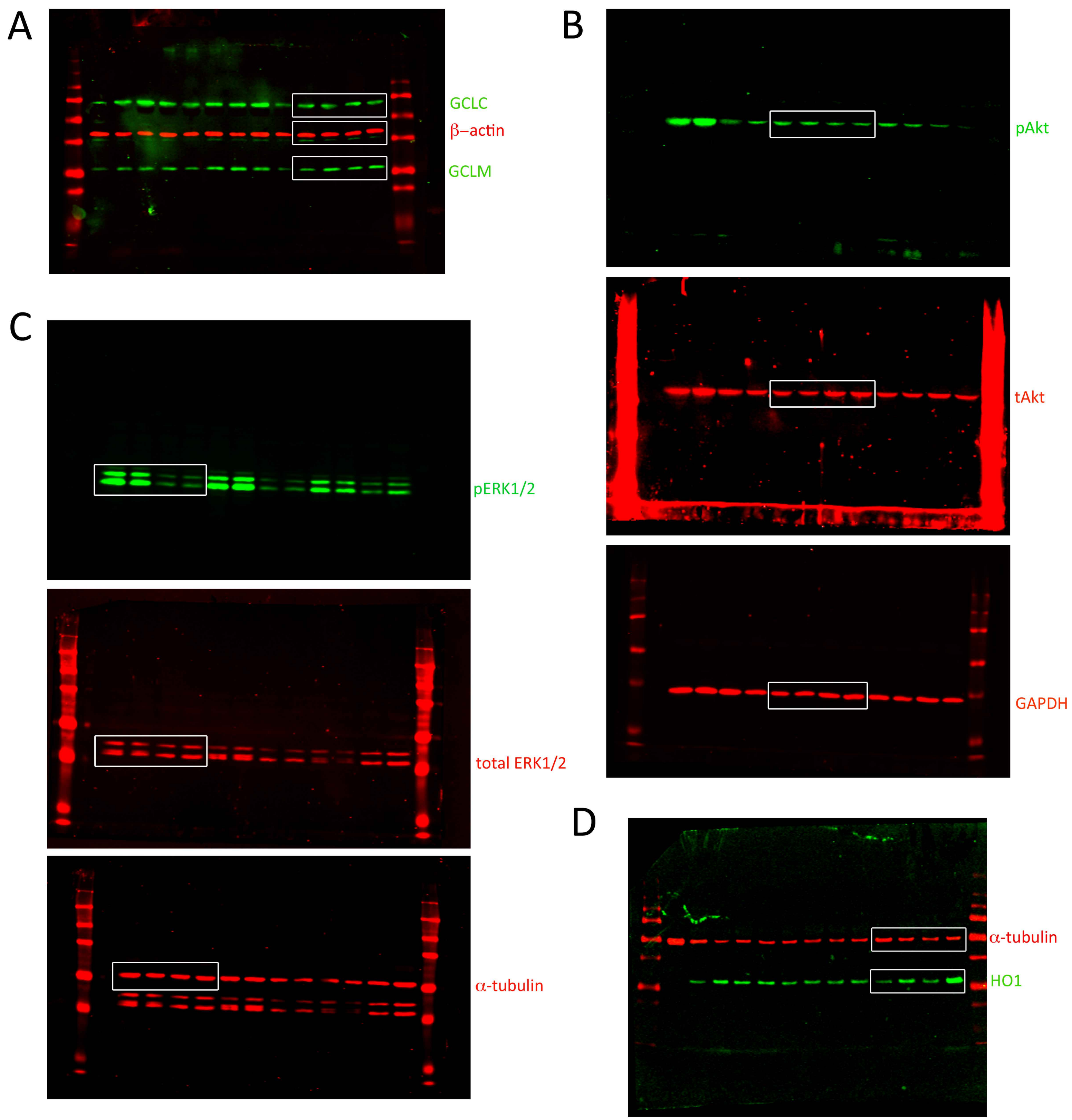
A
GCLC
β–actin
GCLM
B
pAkt
tAkt
GAPDH
C
pERK1/2
total ERK1/2
α-tubulin
D
α-tubulin
HO1

Figure S3. Representative full-length immunoblots for all probings. Full-length immunoblots for (**A**) GCLC and GCLM, (**B**) pAkt and total Akt, (**C**) pERK1/2 and total ERK1/2, and (**D**) HO-1, corresponding to the cropped blots in Figures 3E-F, 3G, 3H, and S2A, respectively. The first lane in (A) and (D) is control adult rat brain lysate. The immunoblot in (A) was first stained for GCLC and β-actin, and then reprobed for GCLM, as the molecular weights are distinct. The immunoblots in (B) and (C) were first stained for pAkt and total Akt or pERK1/2 and total ERK1/2. Next, we re-probed the same blots for the loading controls GAPDH (B) and α-tubulin (C). Note that the signal for total Akt in (B) was dim and the brightness had to be increased to view the bands, which overexposed the molecular weight ladders. In the GAPDH panel, the total Akt bands are too dim to be visible when the GAPDH was not overexposed.
